# Hepatobiliary Adverse Events Associated With the KRAS p.G12C Inhibitor Sotorasib

**DOI:** 10.1002/pds.70104

**Published:** 2025-01-22

**Authors:** Connor Frey

**Affiliations:** ^1^ Department of Medicine University of British Columbia Vancouver British Columbia Canada

**Keywords:** biliary toxicity, hepatotoxicity, kinase inhibitor, *KRAS* G12C, targeted therapy

## Abstract

**Purpose:**

The p.G12C mutation in KRAS is commonly found in many cancers and was previously untreatable until drugs like sotorasib were developed. However, up to 15% of patients treated with sotorasib have experienced hepatobiliary adverse events. To investigate whether these side effects are more common among sotorasib users, a pharmacovigilance study is necessary.

**Methods:**

This study used the FDA adverse event reporting system (FAERS) database, a publicly available repository of reported drug adverse events, and AERSMine, an open‐access pharmacovigilance tool, to investigate these adverse events.

**Results:**

A total of 428 hepatobiliary adverse events were linked to sotorasib. Hepatic cytolysis had the highest reported relative risk at 26.541 and a safety signal of 4.726. Elevated liver and biliary enzymes such as AST, ALT, ALP, and GGT were commonly observed, but with lower reported relative risk and safety signal values, which supports previous real‐world reports.

**Conclusions:**

These findings highlight the hepatobiliary risks associated with sotorasib and underscore the importance of closely monitoring liver function in patients who are using the medication. This is particularly crucial for patients with hepatobiliary cancers, as disease progression and adverse events could be misinterpreted.

**Summary**

- The KRAS p.G12 mutation is common in cancers, with p.G12C being the third most prevalent.
- Up to 15% of patients treated with sotorasib have reported hepatobiliary adverse events.
- Hepatic cytolysis showed the highest reported relative risk (RRR = 26.541) and safety signal (SS = 4.726), indicating a significantly elevated risk.
- Hepatotoxicity, hypertransaminasaemia, and abnormalities in liver function tests, such as elevated AST, ALT, ALP, and GGT, were also significant but had lower reported relative risk and safety signal values.
- Hepatobiliary adverse events should be closely monitored when using sotorasib to treat various hepatobiliary malignancies.

## 1 Purpose

The p.G12 mutation is predominant in human cancers harbouring *KRAS* mutations, with p.G12C being the third most frequently observed [1]. Until recently, these mutations were undruggable, until the development of agents like sotorasib [2]. While now being able to target a canonical *KRAS* mutation, up to 15% of patients have developed hepatobiliary adverse events (AE), highlighting the need to investigate these reports further [2, 3]. Incidence of adverse drug events can be determined in real‐world settings by utilizing large‐scale reporting systems involving a diverse background of patients. Herein, the hepatobiliary AEs of sotorasib are investigated using the US Food and Drug Administration (FDA) Adverse Event Reporting System (FAERS), a US‐based pharmacovigilance database.

## 2 Methods

The FAERS database was accessed through the open‐source tool, AERSMine, where all the data from this study is publicly available [4]. To determine the reported relative risk (RRR), we compared the rate of AEs in patients given sotorasib to the rate of the same events for all other drugs. Higher reported relative risk values indicate a stronger correlation between a specific drug and AE, with values greater than 2.00 being clinically significant. Additionally, we utilized the AERSMine safety signal (SS) to analyze the association between sotorasib and a given AE. We searched for the term *“*sotorasib” as the primary suspect drug with a search period spanning from 2004 Q1 to 2023 Q3. Comparing the adverse events of this drug to those of all drugs across therapeutic areas provides a broad context for its safety profile. This approach establishes baseline risk, aiding in identifying if adverse events are within expected ranges or unusually high. It supports comprehensive safety evaluations, regulatory decision‐making, and detection of novel adverse events, thereby enhancing post‐marketing surveillance.

## 3 Results

In this study, 20 346 289 unique reports were searched, and a total of 532 081 hepatobiliary adverse events were uncovered, with 428 being associated with sotorasib (Table 1). Among the hepatobiliary disorders, hepatic cytolysis showed the highest RRR of 26.541, indicating a significantly elevated risk compared to the general population, with a corresponding SS of 4.726. Hepatotoxicity followed closely with an RRR of 23.203 and an SS of 4.533. Other adverse events such as hepatic function abnormalities, cholestasis, drug‐induced liver injury (DILI), liver disorder, and hepatitis displayed progressively lower RRRs and SS values. In terms of lab investigations, hypertransaminasaemia emerged as a prominent adverse event with a significant RRR of 21.820 and a corresponding SS of 4.445, indicating a substantial elevation in risk compared with baseline. Similarly, individual abnormalities in liver function tests, including elevated levels of aspartate aminotransferase (AST), alanine aminotransferase (ALT), alkaline phosphatase (ALP), and gamma‐glutamyl transferase (GGT), as well as elevated bilirubin, were observed, although with less significant RRR and SS values.

**TABLE 1:** Number of reports for each hepatobiliary adverse event and their associated reported relative risk (RRR) and safety signal (SS).

Adverse event	Total events (*N*)	Sotorasib events (*N*)	RRR^a^, ^b^	SS^c^
Hepatobiliary disorders				
Hepatic cytolysis	5524	29	26.541	4.726
Hepatotoxicity	22 710	53	23.203	4.533
Hepatic function abnormal	43 994	50	11.286	3.495
Cholestasis	19 505	21	10.691	3.417
Drug‐induced liver injury (DILI)	25 061	16	6.332	2.662
Liver disorder	51 573	21	4.041	2.014
Hepatitis	28 316	11	3.855	1.946
Hepatobiliary investigations				
Hypertransaminasaemia	4556	10	21.820	4.445
Liver function test abnormal	19 404	33	16.898	4.076
Elevated aspartate aminotransferase (AST)	66 884	51	7.569	2.919
Elevated alanine aminotransferase (ALT)	76 700	49	6.341	2.664
Elevated alkaline phosphatase (ALP)	33 229	20	5.974	2.578
Elevated gamma‐glutamyl transferase (GGT)	29 553	16	5.373	2.425
Hepatic enzyme elevated	71 326	33	4.591	2.198
Elevated bilirubin	33 318	15	4.468	2.159
Total	531 653	428		

^a^ Relative reporting ratio.

^b^ A RRR > 2.00 is considered clinically significant.

^c^ Safety signal.

## 4 Conclusions

The results of this investigation highlight the range of hepatobiliary abnormalities that can be observed following treatment with sotorasib. As a new agent, sotorasib is being used in patients following multiple lines of pretreatment. In its first major trial, the use of sotorasib caused AST and ALT elevations in 15.1% of patients, ALP was elevated in 7.1%, among more infrequent events including drug‐induced liver injuries, GGT elevation, and abnormal hepatic function, thus in agreeance with this study [2]. When studied in further solid tumours, AST elevation was observed in 13.2% of patients and 11.6% experienced ALT elevation. Grade 3 elevated ALT, AST, and ALP occurred in 4.7%, 2.3%, and 1.6% respectively, with one patient discontinuing treatment due to their elevated ALT and AST [5]. Finally, when studied in pancreatic cancer, 2.6% each developed a drug‐induced liver injury, ALT elevation, AST elevation, and ALP elevation [6].

While not delineated here, multiple reports of hepatotoxicity have been observed for patients who receive sotorasib following immune checkpoint blockade with anti‐PD‐(L)1 therapy. A recent pharmacovigilance study found that when targeted therapies are combined with checkpoint inhibitors, *KRAS*‐directed agents were most strongly associated with the development of hepatitis, with a significant reporting odds ratio of 3.03 [7]. However, it was previously thought that hepatic AEs were rare after sotorasib [8]. However, a recent real‐world study found that 12% of patients developed grade ≥ 3 liver enzyme increase while on therapy [9]. To understand the cause of sotorasib‐induced hepatotoxicity, additional research will be necessary to investigate its underlying pathophysiology. While sotorasib is novel with a smaller number of reports currently, it will undoubtedly gain a larger foothold in the clinic, and a larger set of data will become available to better observe the trends in this study over a broader population and time frame.

## Author Contributions

C.F. was responsible for the collection and analysis of the data, as well as the preparation and review of the final manuscript.

## Ethics Statement

An ethics committee was not involved as the data has already been made publicly available by the United States Food and Drug Administration Adverse Events Reporting System.

## Conflicts of Interest

The author declares no conflicts of interest.
